# Adoption and perception of prescribable digital health applications (DiGA) and the advancing digitalization among German internal medicine physicians: a cross-sectional survey study

**DOI:** 10.1186/s12913-024-11807-1

**Published:** 2024-11-06

**Authors:** Lasse Cirkel, Fabian Lechner, Nadine Schlicker, Jan Leipe, Felix Mühlensiepen, Ivica Grgic, Martin C. Hirsch, Sebastian Kuhn, Johannes Knitza

**Affiliations:** 1 Institute of Artificial Intelligence, University Hospital Gießen-Marburg, Philipps University, Marburg, Germany; 2 Institute for Digital Medicine, University Hospital Gießen-Marburg, Philipps University, Marburg, Germany; 3 Department of Medicine V, Division of Rheumatology, University Medical Center and Medical Faculty Mannheim, Mannheim, Germany; 4 Center for Health Services Research, Faculty of Health Sciences, Brandenburg Medical School Theodor Fontane, Rüdersdorf bei Berlin, Germany; 5 Université Grenoble Alpes, AGEIS, Grenoble, France; 6 Department of Internal Medicine, Division of Nephrology & Institute of Artificial Intelligence, University Hospital Gießen-Marburg, Philipps University, Marburg, Germany

**Keywords:** Digital health applications, DiGA, Digitalization in medicine, Adoption barriers, German healthcare

## Abstract

**Background:**

Therapeutic digital health applications (DiGAs) are expected to significantly enhance access to evidence-based care. Since 2020, German physicians and psychotherapists have been able to prescribe approved DiGAs, which are reimbursed by statutory health insurance. This study investigates the usage, knowledge and perception of DiGAs as well as the growing digitalization among internal medicine physicians in Germany.

**Methods:**

A web-based survey was distributed at the 2024 annual congress of the German Society for Internal Medicine. Participants could respond by scanning a QR code or directly on a tablet.

**Results:**

A total of 100 physicians completed the survey, with a mean age of 43.4 years. The majority were internal medicine physicians (85%). Of the respondents, 31% had already prescribed DiGAs, and 29% had tested one. Self-rated knowledge of DiGAs was low (median score 3.17/10). The main barriers identified were lack of knowledge about effective implementation (60%), lack of time for patient onboarding (27%), and concerns about patient adherence (21%). However, 92% believed that DiGAs could improve care, and 88% expressed interest in specific digital health training. The majority (64%) stated that digitalization had a positive impact on medical care and 39% of physicians expected their daily workload to decrease due to digitalization. In addition, 38% believed that the physician-patient relationship would improve as a result of digitalization.

**Conclusions:**

While physicians widely acknowledged the potential benefits of DiGAs, adoption and understanding remain limited. Specific training in digital health is crucial to accelerate digitalization in internal medicine.

**Supplementary Information:**

The online version contains supplementary material available at 10.1186/s12913-024-11807-1.

## Introduction

The integration of digital technologies into clinical routine, in particular therapeutic digital health applications, offers instant access to evidence-based, personalized treatment. In 2019, a new regulatory framework in Germany has enabled the prescription and reimbursement of approved digital health applications (DiGAs) under the statutory health insurance [1, 2]. The German Federal Institute for Drugs and Medical Devices (BfArM) oversees the evaluation of these applications, ensuring their safety and efficacy, and maintains a library of approved DiGAs [3]. In October 2024, 64 DiGAs have been approved, with the number steadily increasing. These approved DiGAs now include a growing range of internal medicine conditions such as diabetes, hypertension, and heart failure. Additionally, internal medicine physicians can prescribe mental health and orthopedic DiGAs to provide holistic treatment support and help bridge the gap while patients await specialist appointments.

Despite their considerable potential, the successful adoption of DiGAs relies on both healthcare professionals and patients. This challenge reflects a general trend in digital health, where the adoption of new technologies often faces barriers related to user familiarity, system integration issues, and concerns about effectiveness, data security and reliability [4–7]. While previous studies demonstrated the high patient acceptance of DiGAs [8–10], patient acceptance and consent are prerequisites for the effective sharing of personal health information (PHI) through health information exchanges (HIEs) [11]. A study by Busch-Castler et al. found that German patients were willing to share their PHI with HIEs under certain conditions, including perceived information security and a non-commercial organization as the recipient of the PHI [11]. The study also highlighted the importance of face-to-face interactions with physicians in increasing trust in digital health apps and PHI sharing. In contrast, little evidence exists regarding physicians’ usage and perception of DiGAs. Initial studies have focused on general practitioners and rheumatologists and identified implementation barriers such as inadequate reimbursement and poor knowledge [12, 13]. A comprehensive survey conducted in January 2021 examined the experiences of physicians and psychotherapists with DiGAs. However, this study did not address broader perceptions of digitalization in medicine, and its findings on DiGA usage may have shifted due to the rapid pace of digital health advancements [14]. Therefore, the aim of this study was to investigate the usage and perception of DiGAs as well as the increasing digitalization among German internal medicine physicians.

## Methods

### Design and setting

A web-based survey was completed by physicians attending the annual congress of the German Society for Internal Medicine (Deutsche Gesellschaft für Innere Medizin, DGIM) between April 13 and April 16, 2024. Participants could either complete their responses directly on an iPad on-site or scan a QR code to answer the questions anonymously on their own smart devices. The German Society for Internal Medicine is one of the largest European medicine societies with more than 30.000 active members [15]. The Philipps-University Marburg Research Ethics Committee confirmed that no ethical approval was required (24-34 ANZ) for this anonymous survey study.

### Survey

An initial survey draft was created by LC and JK. All authors provided improving comments and approved the final version. The final web-based survey was created using Google Forms and consisted of 16 questions, including 5 questions on personal characteristics (age, gender, work), 5 questions on DiGAs, 5 questions on digitalization in general, and 1 question on whether a dedicated workshop to integrate digital health technologies would be welcomed (translated version, see supplementary file 1).

Specifically, the DiGA-related questions asked whether physicians had ever prescribed or tested a DiGA, how they would rate their own level of knowledge about DiGAs on an 10-point Likert scale (0 = no knowledge, 10 = great knowledge) and what barriers they perceived in prescribing DiGAs. The digitalization-related questions focused on whether digitalization in general improves patient care (5-point Likert scale: 1 = very negative, 5 = very positive), and whether digitalization will reduce or increase physicians’ workload in the future (5-point Likert scale: 1 = my workload will increase due to digitalization, 5 = my workload will decrease due to digitalization), its expected effect on the doctor-patient relationship (5-point Likert scale: 1 = the doctor-patient relationship will significantly worsen due to digitalization, 5 = the doctor-patient relationship will significantly improve due to digitalization), and where they see the potential of digitalization, and what barriers they perceive, including optional free text responses.

### Statistical analysis

The study followed the Checklist for Reporting Results of Internet E-Surveys to guide methodology and presentation of results [16]. Descriptive and summary statistics were used, with frequencies and percentages calculated for categorical variables. Analyses were performed using R version 4.1.0 (R Foundation for Statistical Computing, Vienna, Austria) and Excel Windows (Microsoft Corporation, Redmond, WA, USA).

## Results

### Demographics

A total of 100 participants completed the survey, of whom 51% were male, 48% female and 1% identified as diverse. The mean age was 43.4 years. The majority of physicians (65%) were consultants, with 30% working at university hospitals and 85% in internal medicine (see Table 1 for detailed characteristics).

**Table 1:** Physician characteristics

Characteristics	Respondents, n (%)
Sex	
Female	48 (48.0)
Male	51 (51.0)
Diverse	01 ( 1.0)
Age (years)	
20-30	16 (16.0)
31-40	35 (35.0)
41-50	16 (16.0)
51-60	24 (24.0)
>61	9 (9.0)
Medical Career Stages	
Resident physician	29 (29.0)
Consultant physician	65 (65.0)
Other Designations	6 (6.0)
Specialization	
General Medicine	7 (7.0)
Internal Medicine	85 (85.0)
Other specialties	8 (8.0)
Work environment	
Solo Practice	13 (13.0)
Group Practice	16 (16.0)
Medical Care Center	7 (7.0)
Basic Care Hospital	17 (17.0)
Tertiary Care Hospital	17 (17.0)
University Hospital	30 (30.0)

**Table 2:** DiGA perceptions

Question and answers	n (%)
**Do you think digital health applications can improve care?**	
Yes	92 (92%)
No	8 (8%)
**What do you see as the biggest barrier to the prescription of DiGAs?**^a^	
Little knowledge about implementation	60 (60%)
Lack of time for patient onboarding	27 (27%)
Poor adherence of patients	21 (21%)
Lack of trust in effectiveness	19 (19%)
Lack of adherence of patients	18 (18%)
Little/no remuneration	14 (14%)
Complicated prescription process	12 (12%)
Free text responses^b^	15 (15%)

^a^Multiple answers were possible.

^b^Other barriers mentioned include financial concerns, doubts about effectiveness, technical difficulties as well as problems with communication and use

**Table 3:** Perceived benefits and barriers of increasing digitalization of medicine

Question and answers	n (%)
**What is the biggest advantage of digitalization in medicine for you?**^a^	
Reduction of repetitive activities	68 (68%)
Improved diagnosis/therapy decisions	42 (42%)
Better standardization of clinical decisions (evidence-based medicine)	36 (36%)
More personalized patient care	30 (30%)
More cost-effective patient care	23 (23%)
Free text responses^b^	6 (6%)
**What is the biggest barrier to the general use of digitalization for you?**^a^	
Insufficient knowledge on how to use digital tools	51 (51%)
Data security	46 (46%)
Lack of trust in digital tools	20 (20%)
Fear of the influence of large technology companies	16 (16%)
Responsibility issues when using digital tools	16 (16%)
Fear of digital tools as a new control instance or gold standard that one has to adhere to	15 (15%)
High costs	12 (12%)
Free text responses^c^	3 (3%)

^a^Multiple answers were possible.

^b^Other advantages mentioned include more time with patients, improved patient guidance, higher therapy adherence, and better monitoring and information transfer.

^c^Other barriers mentioned include poor usability, unreliability, and technical problems

### DiGA use and knowledge

Of the physicians surveyed, 31% had previously prescribed DiGAs. Of these, 14% had prescribed DiGAs 10 or more times, with two physicians reporting around 100 DiGA prescriptions. Pearson correlation analysis showed a significant positive correlation between physician age and the number of DiGA prescriptions (r = 0.259, p = 0.009). Perceived knowledge about DiGAs was relatively low, with a median self-rating of 3/10 (IQR: 2-5). There was also a significant positive correlation observed between physician age and their self-rated knowledge of DiGAs (r = 0.378, p<0.001). In addition,the number of DiGA prescriptions was significantly positively correlated with perceived knowledge about DiGAs (r = 0.295, p = 0.003). Overall, 29% of physicians had tested at least one DiGA, and 13% had tested more than one DiGA. 92% of physicians believed that DiGAs had the potential to improve patient care.

### Perceived barriers to DiGAs

The most commonly reported barriers to prescribing DiGAs (Table 2) were lack of knowledge (60%), lack of time to onboard patients (27%), and concerns about patient adherence (21%).

### Perceived impact of digitalization

**Fig. 1:**
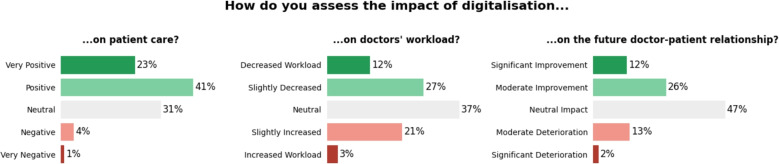
Perceived impact of digitalization of patient care

The majority of physicians (65%) answered that digitalization has had a positive impact on medical care (Fig. 1). In terms of workload, 24% of respondents expected their daily workload to increase as a result of digitalization, while 39% expected it to decrease. In addition, 38% of the respondents believed that digitalization would improve the physician-patient relationship , while 15% believed it would worsen it.

### Perceived benefits and barriers of increasing digitalization of medicine

The main perceived benefits of digitalization were the reduction of repetitive tasks (68%), improved diagnostic and treatment decisions (42%), and better standardization of evidence-based decisions (36%). However, the main barriers were insufficient knowledge of how to use digital tools (51%), concerns about data security (46%), and lack of trust in digital tools (20%) (Table 3). The majority (88%) of physicians expressed interest in specific training on the use of digital tools in clinical practice.

## Discussion

The primary objective of this survey study was to investigate the usage and perception of DiGAs in the context of the increasing digitalization in medicine among German internal medicine physicians. Internal medicine is heavily characterized by the management of chronic diseases, which are associated with significant healthcare costs [17]. While the benefits of lifestyle interventions for these conditions are well established [18], current treatments often remain medication-centric. DiGAs offer a promising tool for enhancing patient self-management, potentially unlocking the full potential of lifestyle interventions to complement conventional treatments. The potential of digital therapeutics is increasingly recognized in other countries, such as Belgium and France, which have also implemented regulatory and reimbursement frameworks to support their adoption [19].

The results revealed a significant discrepancy between physician’s belief in the potential of DiGAs to improve patient care (92%) and their actual usage (32% had previously prescribed a DiGA). A substantial proportion of respondents, particularly those working in hospitals, reported either never or rarely prescribing DiGAs. Several factors are likely to contribute to this low prescription rate with insufficient knowledge, limited trust in the efficacy of DiGAs, and concerns about patient adherence emerging as the primary barriers to adoption.

The observed prescription rate of 31% was lower than in a previous study of general physicians [20], the physician group with the highest prescription rate, followed by orthopaedic surgeons [21]. The lower prescription rate in this study is in line with the results of another survey study among rheumatologists [13]. The observed prescription rate here was 7%, however this study was already published in 2022. This difference may be due to the fact that specialists in secondary care may not consider themselves responsible for treating all conditions in increasingly multimorbid patients. In addition, the availability of DiGAs is currently limited to certain indications, meaning that some specialists may not have access to relevant DiGAs for their primary disease spectrum. Another factor is that many of the physicians in our study worked in hospitals, dealing primarily with inpatients. While DiGA prescriptions are predominantly used in outpatient settings, it is also possible for inpatients. However, the lack of interoperability between hospital software systems significantly hampers the adoption of DiGAs in inpatient care [22].

While physicians were generally positive about the increasing digitalization of medicine, they also expressed concerns about a potential increase in their workload. Lack of time and the high workload were identified as major organizational barriers to adoption [12]. Several other key barriers to widespread implementation of DiGAs were reported, including lack of knowledge, complexity of the prescription process, reimbursement issues and concerns about patient adherence. These findings highlight the urgent need for comprehensive training and education programs to address the knowledge gaps and improve familiarity with digital health tools.

A recent comprehensive mixed-methods study, which included a literature review, supports these findings by identifying low familiarity with digital health solutions as the most commonly cited social barrier, followed by a general lack of awareness [12]. In our survey, a strong majority (88%) of physicians expressed interest in receiving specific digital health training. As previous studies has shown [12], such training is a critical step in overcoming the knowledge gaps identified as the main barrier to DiGA adoption. The development of comprehensive training programs that equip physicians with the necessary knowledge and skills to effectively integrate DiGAs into their practice should be a priority. These training initiatives could be delivered through various formats, such as workshops, online courses, and hands-on training sessions. Professional societies and health care institutions can play a key role in designing and implementing these educational programs, although current efforts remain underutilised [13].

Although this study provides valuable insights, it is important to acknowledge its limitations. As a small cross-sectional study conducted at a single medical conference, the results may not be fully representative of the broader physician population. Additionally, the self-reported nature of the data is also subject to potential bias, and the sample may have been affected by a selection bias, as it is not clear how many physicians declined to participate. Although the conference was specifically dedicated to internal medicine, 15% of the participating physicians were from other specialties. Furthermore, the majority of participants were consultants, representing a narrower subset of the overall physician population. Although the age range of participants was diverse, the mean age was significantly lower than the average age of practicing physicians [23], potentially leading to an overestimation of digital health adoption and affinity [24]. Overall the majority of physicians did however not yet prescribe a DiGA. Future research should aim to validate these findings in larger, more diverse samples and explore the impact of specific interventions such as educational programs, on DiGA adoption rates. Qualitative studies could also provide deeper insights into the specific needs and concerns of different physician subgroups, such as hospital-based versus private practice. Understanding these nuances can inform the development of more targeted support and education programs.

## Conclusion

This survey highlights the implementation gap of DiGAs among German internal medicine physicians. While there is strong confidence in the ability of DiGAs to improve patient care, only one third of respondents had prescribed them. A main barrier appeared to be a lack of knowledge - a challenge that could be effectively addressed through targeted educational programs, which were widely welcomed by internal medicine physicians. This result in in line with a 2024 study where DiGA prescibers were interviewed [25]. This qualitative study also highlighted lack of time and physician reimbursement as major implementation barriers. Addressing these knowledge gaps through comprehensive education is essential to accelerate the adoption of digital health tools in clinical practice [14].

## Supplementary Information

Supplementary Material 1.

## Data Availability

The raw data analysed during the current study are available from the corresponding author upon reasonable request.
