# Supplementary material for: Adoption and perception of prescribable digital health applications (DiGA) and the advancing digitalization among German internal medicine physicians: a cross-sectional survey study
Source: BMC Health Serv Res. 2024 Nov 6;24:1353. doi: 10.1186/s12913-024-11807-1 (PMC11539441; doi:10.1186/s12913-024-11807-1)
Supplement: Supplementary file 1 — Supplementary Material 1. [file 12913_2024_11807_MOESM1_ESM.zip › DGIM_survey.pdf]

DGIM Survey

An anonymous survey by Marburg University Hospital. Thank you for your participation!

Sign in to Google to save your progress. Learn more

* Indicates required question

Which gender do you feel you belong to? *

- [ ] female
- [ ] male
- [ ] diverse

How old are you? (age in years) *

Your answer ______

What is your current position? *

- [ ] Resident physician
- [ ] Specialist physician
- [ ] Other: ______

In which field do you mainly work? *

- [ ] General Medicine
- [ ] Internal Medicine
- [ ] Gastroenterology
- [ ] Endocrinology
- [ ] Pulmonology
- [ ] Emergency Medicine
- [ ] Rheumatology
- [ ] Hematology
- [ ] Other: ____________

Where do you work? (Multiple answers possible) *

- [ ] Private practice
- [ ] Group practice
- [ ] MVZ (Medical care center)
- [ ] Basic care hospital
- [ ] Maximum care hospital
- [ ] University hospital

Next

Clear form

Never submit passwords through Google Forms.

This content is neither created nor endorsed by Google. Report Abuse - Terms of Service - Privacy Policy

Google Forms

DGIM Survey

Sign in to Google to save your progress. Learn more

* Indicates required question

Digital Health Applications and General Information

How often have you prescribed Digital Health Applications (DiGAs)? *

Your answer

How would you rate your Digital Health Applications (DiGAs) knowledge? *

	0	1	2	3	4	5	6	7	8	9	10	
No knowledge	○	○	○	○	○	○	○	○	○	○	○	Very great knowledge

How often have you personally used/tested Digital Health Applications (DiGAs)? *

Your answer

Do you think Digital Health Applications can improve healthcare? *

- ☐ yes
- ☐ no

What do you see as the biggest barrier to prescribing DiGAs? *

- [ ] Little/no remuneration
- [ ] Little knowledge about implementation
- [ ] Lack of trust in effectiveness
- [ ] Complicated prescription process
- [ ] Lack of patient adherence
- [ ] Lack of time for patient onboarding
- [ ] Poor adherence of patients
- [ ] Other: ______

How do you assess the impact of digitalization on patient care? *

	1	2	3	4	5	
Very negative	○	○	○	○	○	Very positive

How do you assess the impact of digitalization on your workload? *

	1	2	3	4	5	
Increased workload	○	○	○	○	○	Decreased workload

How do you assess the impact of digitalization on the future doctor-patient relationship? *

	1	2	3	4	5	
The doctor-patient relationship will deteriorate significantly as a result of digitalisation	○	○	○	○	○	The doctor-patient relationship will be greatly improved by digitalisation

What do you see as the biggest advantage of digitalization in medicine? *

- [ ] More cost-effective patient care
- [ ] Improved diagnosis/therapy decisions
- [ ] Reduction of repetitive activities
- [ ] Better standardization of clinical decisions (evidence-based medicine)
- [ ] More personalized patient care
- [ ] Other: ______

What do you see as the biggest barrier to digitalization for general use? *

- [ ] Data security
- [ ] Responsibility issues when using digital tools
- [ ] Lack of trust in digital tools
- [ ] Fear of digital tools as a new control instance or gold standard that one has to adhere to
- [ ] Fear of the influence of large technology companies
- [ ] Insufficient knowledge on how to use digital tools
- [ ] High costs
- [ ] Other: ______

Would you like training/continuing education on the use of digital tools in clinical practice? *

- [ ] Yes
- [ ] No
- [ ] Don't know

Back Submit Clear form

Never submit passwords through Google Forms.
